# Legume Genetics and Biology: From Mendel’s Pea to Legume Genomics

**DOI:** 10.3390/ijms21093336

**Published:** 2020-05-08

**Authors:** Petr Smýkal, Eric J.B. von Wettberg, Kevin McPhee

**Affiliations:** 1 Department of Botany, Faculty of Sciences, Palacký University, 779 00 Olomouc, Czech Republic; 2 Department of Plant and Soil Sciences and Gund Institute for the Environment, University of Vermont, Burlington, VT 05405, USA; Eric.Bishop-Von-Wettberg@uvm.edu; 3 Plant Sciences and Plant, Pathology Department, Montana State University, Bozeman, MT 59717, USA; kevin.mcphee@montana.edu

**Keywords:** genomics, legumes, nitrogen fixation, proteins

## Abstract

Legumes have played an important part in cropping systems since the dawn of agriculture, both as human food and as animal feed. The legume family is arguably one of the most abundantly domesticated crop plant families. Their ability to symbiotically fix nitrogen and improve soil fertility has been rewarded since antiquity and makes them a key protein source. The pea was the original model organism used in Mendel’s discovery of the laws of inheritance, making it the foundation of modern plant genetics. This Special Issue provides up-to-date information on legume biology, genetic advances, and the legacy of Mendel.

## Introduction

Legumes have always been a part of everyday life, as human food and animal feed, being key protein sources. Legumes represent the second most important family of crop plants after Poaceae (grass family), accounting for approximately 27% of the world’s crop production. While in cereals the major storage molecule is starch, which is deposited in the endosperm, in most of the grain legumes (pulses) the endosperm is transitory and consumed by the embryo during seed maturation. Legume seeds contain a high proportion of proteins (20–40%), and either lipids (soybean, peanut) or starch (or both) as a further carbon source [1]. The importance of legumes for agriculture as well as science has been recognized by the establishment of International Legume Society (ILS) in 2010 (https://www.legumesociety.org), followed by biannual conferences bringing together people working on broad aspects of legume biology. The last ILS conference was held in 2019 in Poland and this Special Issue has been made to reflect some of the presented work. The long-term strategy of ILS is linking together the different aspects of agricultural research on grain and forage legumes worldwide.

The Fabaceae is the third-largest family of flowering plants, with over 800 genera and 20,000 species. Currently, three major groups are recognized and regarded as subfamilies: the mimosoid legumes, Mimosoideae (sometimes regarded as the family Mimosaceae with four tribes and 3270 species); the papilionoid legumes, Papilionoideae (or the family Fabaceae/Papilionaceae with 28 tribes and 13,800 species); and the caesalpinioid legumes, Caesalpinoideae (or the family Caesalpiniaceae with four tribes and 2250 species) [2]. It is an extremely diverse family with a worldwide distribution, from arctic-alpine herbs to annual xerophytes and forest trees.

Legumes have played an important part in cropping systems since the dawn of agriculture. Records from the oldest civilizations of Egypt and eastern Asia demonstrate the ancient use of various beans, peas, vetches, soybeans, and alfalfa. One of the early Greek botanists, Theophrastus, in the third century before Christ, wrote of leguminous plants “reinvigorating” the soil and stated that beans were not a burdensome crop to the ground but even seemed to manure it. The Romans emphasized the use of leguminous plants for green manuring; they also introduced the systematic use of crop rotations, a practice that was forgotten for a time during the early Middle Ages and partly also in today´s agricultural practice.

Members of the Fabaceae were domesticated as grain legumes in parallel with cereal domestications [3,4,5,6,7,8]. There are 13 genera (in six legume tribes) that constitute major legume crops [1,2]. Among the first legumes to be domesticated were members of the galegoid tribe such as peas, faba beans, lentils, grass peas and chickpeas, which arose in the Fertile Crescent of Mesopotamian agriculture. These grain legumes (pulse legumes) accompanied cereal production and formed important dietary components of early civilizations in the Near East and the Mediterranean regions. Similar domestications of *Phaseolus* in the New World and *Glycine* in East Asia have had similar importance for human dietary diversity and security.

Cultivated legumes fulfill many human needs beyond being directly consumed by people. Many tree-sized species in the legume family are valuable for their hard, durable timber. Species from the genera *Aeschynomene*, *Arachis*, *Centrosema*, *Desmodium*, *Macroptilium*, and particularly *Stylosanthes* offer promise for improved tropical pasture systems. The barks of some species of acacias (*Acacia dealbata*, *A. decurrens*, and *A. pycnantha*) are sometimes used as sources of tannins, chemicals that are mostly used to manufacture leather from animal skins. Some important dyes are extracted from species in the legume family. One of the world’s most important natural dyes is indigo, extracted from the foliage of the indigo (*Indigofera tinctoria*) of south Asia and to a lesser degree from American indigo (*I. suffruticosa*) of tropical South America. Derris or rotenone is a poisonous alkaloid extracted from *Derris elliptica* and *D. malaccensis* that has long been used by indigenous peoples of Southeast Asia as arrow and fish poisons. Rotenone is now used widely as a rodenticide to kill small mammals and as an insecticide to kill pest insects. Fenugreek (*Trigonella foenum graecum*), the seeds of which are used as a spice in curries. Legumes include also valuable fiber plants, such as the sunn-hemp of India (*Crotalaria juncea*) and Hemp sesbania (*Sesbania exaltata*) used by the Indians of the southwestern United States. Some legumes such as licorice (*Glycyrrhiza glabra*) and goatsrue (*Tephrosia virginiana*) have medicinal value; many others rank among ornamental plants (for example *Lathyrus odoratus*), and legumes are of great importance for honey production.

The pea (*Pisum sativum* L.) was the original model organism used in Mendel´s discovery (1866) of the laws of inheritance, making it the foundation of modern plant genetics. It had already been an object of experimental work before Mendel [9,10]. Despite their close phylogenetic relationships, crop legumes differ greatly in their genome size, base chromosome number, ploidy level, and reproductive biology. To establish a unified genetic system for legumes, two legume species in the Galegoid clade, *Medicago truncatula* and *Lotus japonicus*, from Trifolieae and Loteae tribes, respectively, were selected as model systems for studying legume genomics and biology [11,12]. Now, many legume crops have well-studied genetic systems. In a few cultivated legumes, comprehensive genetic analysis is limited due to the large size of their genomes. For soybeans, the most widely grown and economically important legume, a genome has been available since 2010 [13]. For the common bean (*Phaseolus vulgaris*), the most widely grown grain legume, a genome has been available since 2014 [14]. Many more legumes have been sequenced since. These genome sequences are now completed by a broad range of genomic resources, including tools for genome-wide association studies, diversity panels, and online databases [15]. These tools facilitated increasingly widespread efforts to implement molecular breeding in legumes. The existence of reference genomes is fundamental for the advancement of genetic mapping approaches using either classical biparental population or association mapping on wider panels. This has been shown in several papers in this issue [16,17] for soybean. Having genome-wide data on diversity on a sufficiently large and diverse set of accessions, along with accumulated phenotypic trait descriptions, provides the tools to conduct genome-wide association studies and genomic selection. This either might lead to the identification of candidate loci/genes governing studied traits or provide useful markers applicable for breeding [18,19].

The history of legume crop domestication is not only of theoretical interest to provide insight into evolution but also can be used in breeding of recently domesticated crops, as shown in lupine [20] and potentially applied to a broader range of crop wild relatives. Legumes are particular among the plant species in their ability to fix atmospheric nitrogen. Owing to their biology including symbiotic nitrogen fixation, legumes are vital components of sustainable agriculture. This has been acknowledged in all cropping systems. Although the fundamentals of bacteria and host plant symbiosis have been elicited, there are still numerous aspects to be studied, such as allelic variation of identified genes, as shown on red clover [21].

Since most of the legume crops are used as food or feed in form of mature, dry seeds, their nutritional composition is of great importance. The study of Sivasakthi et al. [22] shows an elegant application of basic knowledge of one of the genes underlying a classical Mendelian trait, green cotyledons, identified and applied in chickpea. Seed composition can be altered by water availability or other abiotic stresses, as shown in studies of lupine seeds [23]. Similarly, dissection of the molecular mechanisms of resistance to biotic and abiotic are of high relevance both in order to understand evolutionary mechanisms between pathogens/triggers and hosts as well as to facilitate the breeding process. Mutant lines are helpful in elucidation of gene function, as shown in soybeans [24]. Since pathogens display high variation potential and are able to quickly overcome single gene/allele resistance, it is important to identify the allelic variation of a given gene, as shown in powdery mildew resistance in peas [25]. Climate change is already impacting all crops including legumes. There is a great need to understand the mechanisms of stress avoidance/tolerance/resistance to minimalize this impact. The review of Kumar et al. [26] offers a view on breeding climate-resilient legume crops, which is vital particularly for tropical and subtropical countries already facing scarcity of water and soil resources. In current biology, there are commonly integrated various approaches in order to study complex biological pathways, such as that shown by the study of lupine flower development [27]. This work combines genomic, transcriptomic, and small RNA sequencing to understand the process of lupine flower ablation. Owing to progress in genomic methods such as next-generation sequencing, genetics and genomics is not limited to model species and is being applied to any species including crops with complex, polyploid genomes [28]. Evolutionary scenarios of speciation are a recurrent theme in biology, and especially in plants, there are often various pathways to speciation, including frequent hybridization and polyploidy. A central aspect of speciation is the establishment of gene-flow barriers. One of the ways to do this is the interaction between plastid and nuclear genomes leading to either viable to inviable progeny. In peas, the interaction between the chloroplast and nuclear-encoded genes results in either normal or albino/chlorotic plants. The study of Nováková et al. [29] shows the variation of respective genes in natural pea populations as well as identifying the influence of a domestication-imposed bottleneck.

Although Mendel’s peas were the first “model” plant, legume biology has long lagged behind more successful models from the Brassicaceae family or economically important cereals. For Borlaug, grain legumes were the “slow runners” of the green revolution because of the limited extent to which they saw the genetic gains that have characterized breeding of cereals for the past century. However, owing to progress in genomic and phenotyping technologies together with recognition of their importance for ecology of natural or agronomical systems, they are gradually gaining ground. We look for seeing new work in legumes, including releases around the world of new legume varieties bred with genomic resources.
